# Supplementary material for: The effects of genital myiasis on the diversity of the vaginal microbiota in female Bactrian camels
Source: BMC Vet Res. 2022 Mar 5;18:87. doi: 10.1186/s12917-022-03189-5 (PMC8897907; doi:10.1186/s12917-022-03189-5)
Supplement: Supplementary file 5 — Additional file 5. [file 12917_2022_3189_MOESM5_ESM.zip › MPL201709200_16s_yy/Treat1/css/js_css/jsPDF-master.zip!/jsPDF-master/docs/plugins_autoprint.js.html]

plugins/autoprint.js - Documentation


## Home

### Classes

- jsPDF

### Global

- addFont
- addHTML
- addMetadata
- addPage
- autoPrint
- CapJoinStyles
- circle
- ellipse
- getFontList
- lines
- lstext
- output
- rect
- roundedRect
- save
- setDisplayMode
- setDrawColor
- setFillColor
- setFont
- setFontSize
- setFontStyle
- setLineCap
- setLineJoin
- setLineWidth
- setPage
- setProperties
- setTextColor
- text
- triangle

# plugins/autoprint.js

```
/**
 * jsPDF Autoprint Plugin
 *
 * Licensed under the MIT License.
 * http://opensource.org/licenses/mit-license
 */

 /**
 * Makes the PDF automatically print. This works in Chrome, Firefox, Acrobat
 * Reader.
 *
 * @returns {jsPDF}
 * @name autoPrint
 * @example
 * var doc = new jsPDF()
 * doc.text(10, 10, 'This is a test')
 * doc.autoPrint()
 * doc.save('autoprint.pdf')
 */

(function (jsPDFAPI) {
	'use strict';

	jsPDFAPI.autoPrint = function () {
		'use strict'
		var refAutoPrintTag;

		this.internal.events.subscribe('postPutResources', function () {
			refAutoPrintTag = this.internal.newObject()
				this.internal.write("<< /S/Named /Type/Action /N/Print >>", "endobj");
		});

		this.internal.events.subscribe("putCatalog", function () {
			this.internal.write("/OpenAction " + refAutoPrintTag + " 0" + " R");
		});
		return this;
	};
})(jsPDF.API);
```

  

Documentation generated by JSDoc 3.4.2 on Sun Oct 09 2016 11:08:27 GMT+0100 (BST) using the docdash theme.
